# Weight loss in the early stage of progressive supranuclear palsy

**DOI:** 10.1002/brb3.616

**Published:** 2016-12-05

**Authors:** Ayako Tsuge, Satoshi Kaneko, Reika Wate, Mitsuaki Oki, Masato Nagashima, Shinya Asayama, Masataka Nakamura, Kengo Fujita, Akemi Saito, Norihiro Takenouchi, Hirofumi Kusaka

**Affiliations:** ^1^ Department of NeurologyKansai Medical UniversityHirakataOsakaJapan; ^2^ Department of MicrobiologyKansai Medical UniversityHirakataOsakaJapan

**Keywords:** movement disorders, neurodegenerative disorders, Parkinson's disease, progressive supranuclear palsy

## Abstract

**Objectives:**

To clarify whether weight change in patients with Parkinson's disease (PD) or progressive supranuclear palsy (PSP) is caused by the disease itself or secondarily by other factors.

**Materials and Methods:**

We conducted a retrospective analysis of 51 patients with PD and 14 patients with PSP, especially during the early stage of their diseases. All patients were independent in terms of their activities of daily living and did not have any feeding difficulty.

**Results:**

The body mass index measured within 3 years after the disease onset did not show a significant difference between the two diseases. However, the subsequent weight was stable in patients with PD and significantly decreased in patients with PSP.

**Conclusions:**

Weight loss begins in the early stage of PSP, whereas dopaminergic treatment may contribute to keep weight in the early stage of PD through reduction of energy expenditure and/or improvement in appetite.

## 1 Introduction

Weight loss in patients with neurodegenerative diseases is frequently observed and affects the patients' prognosis. A cause‐related and patient‐tailored treatment can improve the quality of life (Aziz et al., 2008), but the underlying pathomechanism of weight loss is multifactorial. Whether this weight loss is primarily due to the disease itself or is secondarily caused by decreased food intake, depression, dysphagia, dysfunctions in the gastrointestinal system, or side effects of medication has not been well investigated. Changes in the body mass index (BMI) of patients with dysphagia due to Parkinson's disease (PD) or progressive supranuclear palsy (PSP) have been reported (Jankovic, Wooten, Van der Linden, & Jansson, 1992), but these patients were in an advanced stage of their disease, and their pathophysiology was complex. Moreover, the time course of weight changes or comparison of such change among other neurodegenerative diseases presenting as parkinsonism has never been reported.

Here, we report a comparative study on weight change in patients with PD or PSP, and discuss the pathophysiology of the weight change in these neurodegenerative diseases. To exclude the complex pathomechanism at work in advanced stages, we focused on the early stages of these diseases.

## 2 Patients and Methods

Patients with PD or PSP in our outpatient departments were enrolled in this study. All subjects gave their written informed consent to this study, which was approved by the local ethics committee. Clinical diagnoses were made according to the established diagnostic criteria for PD (Hughes, Daniel, Kilford, & Lees, 1992) and PSP (Litvan et al., 1996).

Weight can be influenced by many factors, and therefore we focused on the early stages of these diseases and set up criteria for disease duration: (1) the first measurement of weight was made within 3 years from the onset of extrapyramidal symptoms and (2) the interval between the first and the second measurements of weight was within 3 years. According to these criteria, we conducted a retrospective study. All patients were independent in terms of activities of daily living up to the second measurement of weight. To exclude the possibilities of other factors affecting the body weight, patients with dysphagia treated by percutaneous endoscopic gastrostomy and those who had a past medical history of endocrine‐metabolic disease, malignancy, depression, bone fracture, or orthopedic surgery were excluded. As serotonin can affect the appetite and body weight, patients treated with antidepressants such as selective serotonin reuptake inhibitors (SSRI) and/or serotonin norepinephrine reuptake inhibitors (SNRI) were also excluded.

Clinical diagnosis, gender, age of disease onset, date of first visit, and medication were recorded on the entry sheets. All patients with PD were evaluated with the Unified Parkinson's Disease Rating Scale (UPDRS). All patients with PSP were evaluated with PSP rating scale (PSPRS; Golbe & Ohman‐Strickland, 2007). Doses of anti‐parkinsonian drugs were converted to their levodopa equivalent dose (LED) according to a previously reported conversion formula (Tomlinson et al., 2010).

The first weight, BMI and the second weight were abbreviated as BW_1_ (kg), BMI_1_ (kg/m^2^) and BW_2_ (kg), respectively. As the BMI can be influenced by a forward bend posture or by osteoporosis, the change in weight was evaluated as that in the weight itself, which was calculated as ∆BW (=BW_2_ − BW_1_).

Data were analyzed as follows:

1. BMI_1_ values were compared between PD and PSP patients.
2. ∆BW was evaluated for each disease.

Statistical analysis of BMI_1_ was done by performing the Mann–Whitney test and that of ∆BW, by use of the paired‐*t* test and Wilcoxon test. Statistical significance was set at *p* < .05.

## 3 Results

Profiles of the patients are summarized in Table 1. All patients with PSP were diagnosed as Richardson type based on the criteria. Mean ages at the onset of disease were 67.1 ± 7.4 (mean ± *SD*) years for PD and 69.4 ± 6.3 for PSP. Disease duration at the first measurement of weight was 1.4 ± 0.8 years for PD and 1.7 ± 1.0 for PSP, and there was no statistically significant variation between them (Mann–Whitney test).

**Table 1:** Profiles of patients with Parkinson's disease (PD) and progressive supranuclear palsy (PSP)

	PD			PSP			*p* Value		
	Total	Male	Female	Total	Male	Female	Total	Male	Female
Number of patients	51	19	32	14	8	6			
Age at onset (years)	67.1 ± 7.4	67.3 ± 6.4	67.0 ± 8.0	69.4 ± 6.3	72.3 ± 5.6	65.4 ± 5.0	.243	.055	.645
Age at BW_1_ (years)	68.5 ± 7.3	68.9 ± 6.6	68.3 ± 7.8	71.0 ± 6.4	74.0 ± 6.0	67.1 ± 4.9	.240	.056	.733
Age at BW_2_ (years)	70.3 ± 7.5	70.8 ± 6.7	70.0 ± 7.9	72.8 ± 6.4	75.6 ± 6.2	69.1 ± 4.9	.210	.104	.968
Disease duration at BW_1_ (years)	1.4 ± 0.8	1.6 ± 0.7	1.2 ± 0.8	1.7 ± 1.0	1.6 ± 1.0	1.7 ± 1.1	.281	.915	.279
Disease duration at BW_2_ (years)	3.2 ± 1.1	3.5 ± 1.0	3.0 ± 1.0	3.4 ± 1.9	3.3 ± 1.9	3.5 ± 2.0	.719	.631	.470
LED at BW_2_ (mg)	243.7 ± 145.6	289.6 ± 121.5	217.9 ± 153.3						

Values are mean ± standard deviation. LED, levodopa equivalent dose. BW_1_, BW_2_, body weight at the first and the second measurement.

*p* Value of variables comparing PD and PSP.

Some patients were already being medicated for parkinsonism at the time of the first measurement: two male patients (LED, 370.8 ± 5.9 mg) and two female patients (LED, 75.0 ± 35.4 mg) with PD, one male patient (LED, 200 mg) with PSP. At the time of the second measurement, medication was being given to 18 male patients (LED, 289.6 ± 121.5 mg) and 29 female patients (LED, 217.9 ± 153.3 mg) with PD and to 2 male patients (LED, 250 ± 71 mg) with PSP. At the time of the second measurement, 30 patients with PD were treated with dopamine agonists (19 patients treated with pramipexole and 11 patients treated with ropinirole). None of patients with PSP was treated with dopamine agonists. According to the medical records and patients interview, all patients did not have any symptoms of depression, apathy, anorexia, or hyperphagia.

The mean UPDRS Part III scores of the patients with PD at the time of BW_1_ and BW_2_ were 23.2 ± 9.5 and 17.7 ± 7.8, respectively. The mean PSPRS scores for bulbar symptoms at the time of BW_1_ and BW_2_ were 0.8 ± 0.4 and 1.5 ± 0.5, respectively. All patients did not have difficulty in dietary intake at the time of BW_1_ and BW_2_.

BW_1_, BW_2_, BMI_1_, ∆BW, and intervals between the first and the second measurements are indicated in Table 2. BMI_1_ values, which were measured in the very early stage and thus under little effect of medication, were 22.3 ± 3.4 kg/m^2^ for PD and 23.2 ± 3.2 kg/m^2^ for PSP.

**Table 2:** Results of patients with Parkinson's disease (PD) and progressive supranuclear palsy (PSP)

	PD			PSP			*p* Value		
	Total	Male	Female	Total	Male	Female	Total	Male	Female
BW_1_ (kg)	54.3 ± 8.5	58.9 ± 5.7	51.7 ± 8.8	58.6 ± 8.0	60.2 ± 9.1	56.5 ± 6.3			
BMI_1_ (kg/m^2^)	22.3 ± 3.4	22.2 ± 2.6	22.4 ± 3.8	23.2 ± 3.2	22.5 ± 3.9	24.2 ± 2.0			
BW_2_ (kg)	53.8 ± 8.6	58.2 ± 6.9	51.3 ± 8.6	51.3 ± 9.2	54.5 ± 9.8	47.1 ± 6.8			
BMI_2_ (kg/m^2^)	22.4 ± 3.5.7	22.3 ± 3.1	22.4 ± 3	20.7 ± 3.3	20.9 ± 3.9	20.6 ± 2.8			
ΔBW (kg)	−0.5 ± 3.0	−0.7 ± 3.4	−0.4 ± 2.9	−7.3 ± 6.5	−5.6 ± 5.7	−9.4 ± 7.3	<.001	.007	.003
Interval between BW_1_ and BW_2_ (years)	1.8 ± 0.8	1.9 ± 0.7	1.7 ± 0.8	1.7 ± 1.0	1.7 ± 1.0	1.8 ± 1.0	.695	.471	.904

Values are mean ± standard deviation. BW_1_, BW_2_, body weight at the first and the second measurement. BMI_1_, BMI at the first measurement. ΔBW, BW_2_ − BW_1_.

*p* Value of variables comparing PD and PSP.

**Figure 1:**
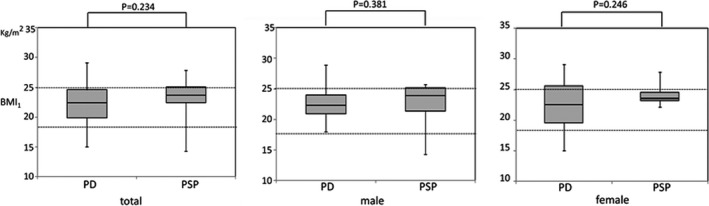
Comparison of BMI_1_ between Parkinson's disease (PD) and progressive supranuclear palsy (PSP). Dotted lines indicate the normal range of BMI according to WHO criteria. Each box plot shows the median, 25th and 75th percentiles. Whiskers indicate the 5th and 95th percentiles

Body mass index in the range from 18.5 to 25 is classified as “normal weight” according to WHO criteria. Therefore, 65% of PD and 64% of PSP patients were categorized as having a “normal weight” in this study (Figure 1). No statistical differences among BMI_1_ of PD and PSP were observed (Mann–Whitney test).

**Figure 2:**
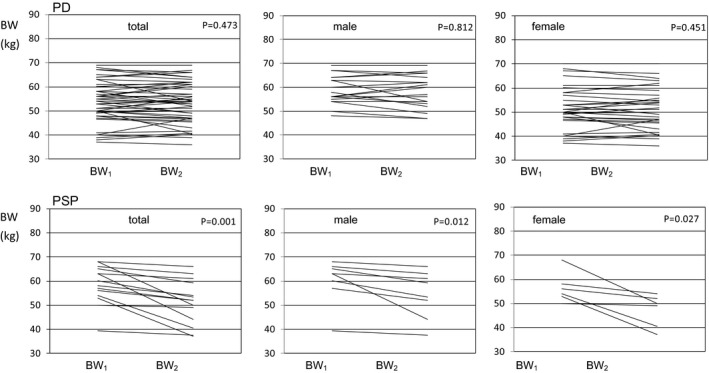
Weight change in Parkinson's disease (PD) and progressive supranuclear palsy (PSP). Lines indicate the weight change between BW_1_ and BW_2_ of individual patients

Measurement intervals for ∆BW were 1.8 ± 0.8 years for PD and 1.7 ± 1.0 for PSP, and no statistically significant difference between these intervals was observed (Mann–Whitney test). For PD patients, their ∆BW was −0.5 ± 3.0, indicating no statistically significant difference. However, in the case of PSP patients, ∆BW was −7.3 ± 6.5 kg, which was a statistically significant decrease in weight. The decrease in weight was statistically significant for total, male, and female patients with PSP (<.05; Figure 2).

## 4 Discussion

We investigated the change in weight of patients with PD and PSP in the early stage of their diseases. Comparison of the BMI_1_ measured within 3 years after disease onset did not show any significant difference between PD and PSP. However, the temporal change in weight was obviously different between these patients; that is, there was a significant decrease in the weight of PSP patients, whereas the weight of the PD patients remained stable, showing no significant change.

Loss of weight as a nonmotor symptom of PD has been previously mentioned in several reports. Patients with a longer medical history of the disease show a greater decrease in the mass of body fat (Sakajiri & Takamori, 1997). Also, triceps skin fold thickness, which indicates the calorie reserves stored in the form of fat, was reported to be significantly decreased in patients with advanced PD (Beyer, Palarino, Michalek, Busenbark, & Koller, 1995). Therefore, weight loss in PD patients, especially in advanced stage, may be attributed mainly to the loss of fat, and not to that of muscle. However, loss of muscle volume due to sarcopenia and/or frail frequently appears in advanced stage of PD and PSP. It has not yet been settled whether the main cause of this reduction in fat or muscle is primarily due to the disease itself or is secondarily caused by reduced food intake or side effects of medication.

A change in weight is thought to result from a disturbed balance between energy intake and energy expenditure (Aziz et al., 2008). Decreased energy intake can be caused by disturbed olfaction or taste, depression, dysphagia, eating difficulty due to parkinsonism, slowing of gastric emptying time, decreased motility of the gastrointestinal tract, or impaired absorption (Pfeiffer, 2003). Especially, dysphagia severely affects the amount of food intake. The BMI of PD patients with dysphagia was earlier reported to show a significantly greater decrease than that of PD patients without dysphagia (Nozaki, Saito, Matsumura, & Miyai, 1999). In this study, it is noteworthy that the weight of patients with PSP in its early stage decreased, in spite of exclusion of patients suffering feeding difficulty. An increase in energy expenditure can be caused by resting tremor or muscle rigidity. PD and PSP patients have, more or less, increased energy expenditure due to tremor and/or rigidity (Levi, Cox, Lugon, Hodkinson, & Tomkins, 1990). However, we could not analyze the source of the disturbance in energy balance in relation to weight change in this study.

Dopaminergic treatment is reported to be associated with decreased weight in patients with advanced PD (Bachmann, Zapf, Brunner, & Trenkwalder, 2009). In this study, we focused on the early stages of PD and PSP, and medication had not been started at the time BW_1_ was measured in almost all of the patients. However, almost all of the PD patients were treated with anti‐parkinsonian drugs by the time that BW_2_ was determined. Therefore, there is a possibility of the protective effect of medication on the weight of the PD patients, who maintained a stable weight. Appropriate treatment of tremor and rigidity in the early stages of PD might have reduced the energy consumption. Appetite of patients with PD might have been improved by dopaminergic treatment (Aiello, Eleopra, & Rumiati, 2015). On the other hand, since most of PSP patients have not received dopamine treatment, it is not likely that medication has affected their change in weight.

In the early stages of PD and PSP, the energy expenditure, may be different and mainly affect the change in weight. Energy expenditure is supposedly promoted in PSP. Reduction of energy expenditure and/or improvement in appetite by dopaminergic treatment may contribute to keep weight in the early stages of PD. Hypothalamic neuropeptides, which control the general metabolism; leptin, an adipose tissue‐derived hormone that regulates body‐fat mass; and disease‐specific neuropathological changes in the hypothalamus, should be investigated in a future study.

## Conflict of Interest

The authors have no conflict of interest to report.
